# Revisiting pathologic myopia: imaging evidence of an inflammatory component in the pathogenesis of myopic degeneration

**DOI:** 10.3389/fmed.2026.1745948

**Published:** 2026-01-21

**Authors:** Alex Fonollosa, Joseba Artaraz

**Affiliations:** 1 Department of Ophthalmology, Biocruces Bizkaia Health Research Institute, Cruces University Hospital, University of the Basque Country, Barakaldo, Spain; 2 Department of Retina, Instituto Oftalmológico Bilbao, Bilbao, Spain

**Keywords:** intraocular inflammation, lacquer cracks, myopic degeneration, patchy atrophy, pathologic myopia, uveitis

## Abstract

Pathologic myopia has traditionally been viewed as a degenerative disorder caused by mechanical stretching and choroidal ischemia. However, converging clinical, molecular, and imaging data increasingly suggest that chronic low-grade inflammation contributes to both the onset and progression of myopic retinal degeneration. Recent studies have identified inflammatory patterns—including multifocal choroiditis/punctate inner choroidopathy (MFC/PIC)–like lesions, periatrophic inflammatory “plumes,” and secondary Multiple Evanescent White Dots Syndrome (MEWDS)—often localized at sites of retinal pigment epithelium–Bruch’s membrane disruption. Parallel laboratory evidence indicates dysregulation of cytokines, activation of the complement cascade, and engagement of intracellular signaling pathways such as JAK–STAT within the myopic eye. Together, these findings support a model in which mechanical stress and hypoxia act as triggers for sustained immune activation, promoting extracellular-matrix remodeling, choroidal thinning, and progressive atrophy. Recognizing inflammation as an integral component of the pathophysiology of pathologic myopia may open new therapeutic perspectives, including immunomodulatory or complement-targeting approaches.

## 1 Introduction

Myopia has reached epidemic proportions, with projections indicating that nearly half of the global population will be myopic by 2050 and around 10% will have high myopia (HM) (1). Pathologic myopia (PM) is defined as excessive axial elongation associated with myopia that leads to structural changes in the posterior segment of the eye (including posterior staphyloma, myopic maculopathy, and high myopia-associated optic neuropathy) that can lead to loss of best-corrected visual acuity (2). Despite advances in our understanding of biomechanical stretching and choroidal hypoperfusion, mounting evidence supports an additional pathogenic axis in PM: chronic, subclinical inflammation within the eye. It has been suggested that inflammatory processes and scleral elongation appear to be linked through a self-perpetuating feedback loop. Low-grade inflammation can precede mechanical changes by activating cytokines and matrix metalloproteinases that soften and thin the sclera, facilitating axial elongation. In turn, stretching of ocular tissues induces hypoxia and release of damage associated molecular patterns (DAMPs), further amplifying local immune activation. Thus, inflammation is both a potential trigger and a consequence of scleral remodeling in PM (3–5). On the other hand, the association of myopia with certain types of posterior uveitis like multifocal choroiditis/punctate inner choroiditis (MFC/PIC) has largely been known. Interestingly, in a few clinical studies it has been proposed that tissue degeneration (specifically Bruch’s membrane ruptures) may trigger the development of clinically evident inflammatory lesions (6) and that these lesions may not only contribute to but also be a key driver of atrophy progression in PM (7, 8), supporting the concept of a feedback mechanism whereby degeneration triggers, and is exacerbated by, inflammation.

Here we review the published multimodal imaging features of inflammatory lesions observed in PM and present some of our own cases, strengthening the concept of the relevance of inflammatory processes in the pathogenesis of PM.

## 2 MFC/PIC lesions as precursors of patchy atrophy, and lacquer cracks as precursors of MFC/PIC lesions

**Figure 1:**
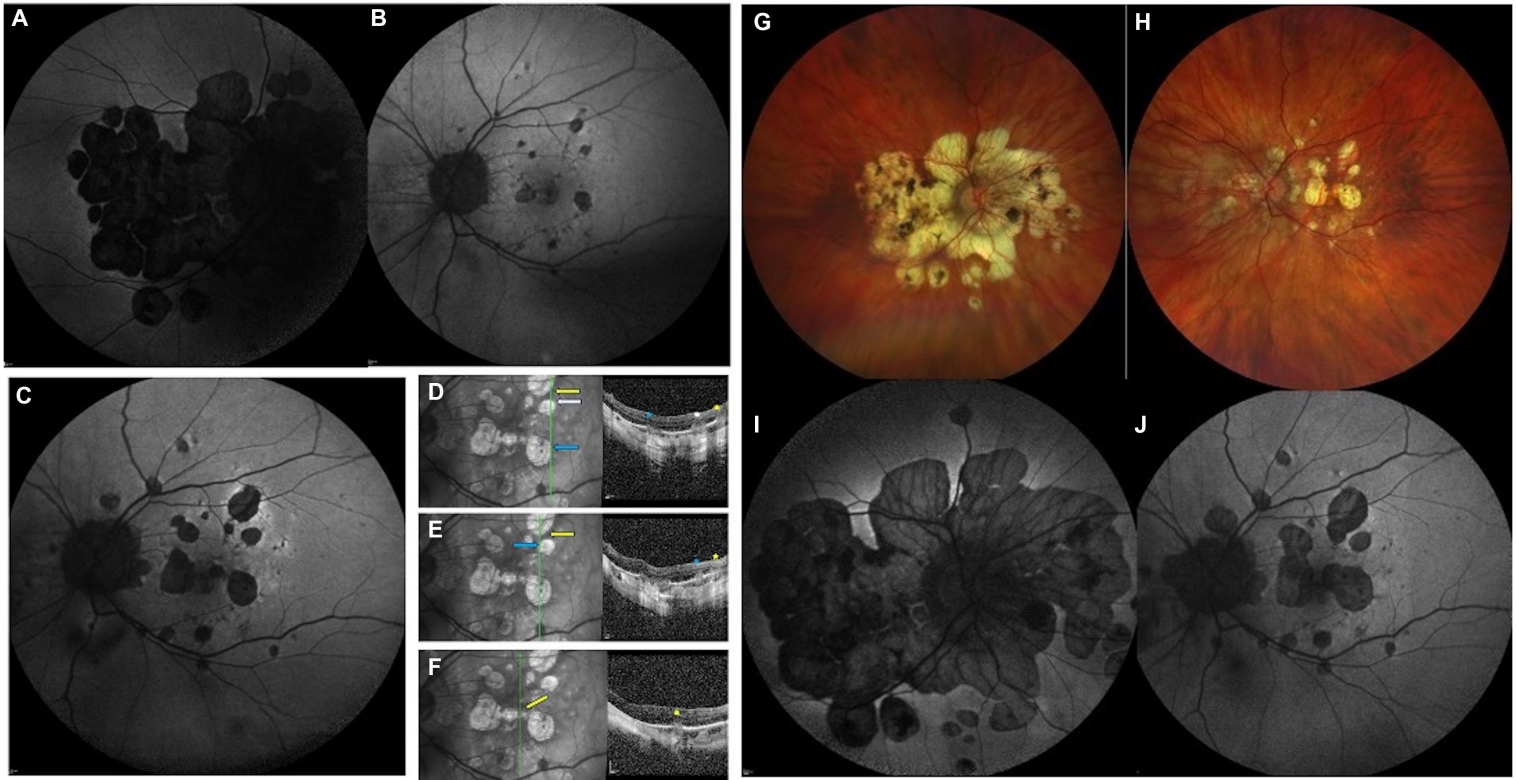
Highly myopic woman (−11 D OU) diagnosed with punctate inner choroiditis (PIC) in the left eye (LE) at the age of 37. According to her medical history, she had experienced an inflammatory episode in the right eye (RE) at the age of 15, which was not diagnosed at that time. Panels **(A,B)** show fundus autofluorescence (FAF) at baseline, when PIC was diagnosed. The patient was treated with corticosteroids and immunosuppressive agents. Despite therapy, several documented inflammatory flares occurred over the years, resulting in progressive enlargement of atrophic lesions. It is likely that the previously undiagnosed inflammatory process in the RE was also PIC, with subsequent atrophy expansion over time. Panel **(C)** shows FAF from one of the documented flares in the LE, 5 years after the initial diagnosis of PIC, with a hyperautofluorescent halo surrounding hypoautofluorescent lesions. Panels **(D–F)** show OCT scans revealing typical acute inflammatory lesions (asterisks); the location of each lesion in the corresponding fundus image is indicated by arrows. Panels **(G–J)** show retinography **(G,H)** and FAF **(I,J)** obtained at the age of 47. Patchy atrophy is evident, more severe in the RE, with enlargement of the atrophic areas over time.

**Figure 2:**
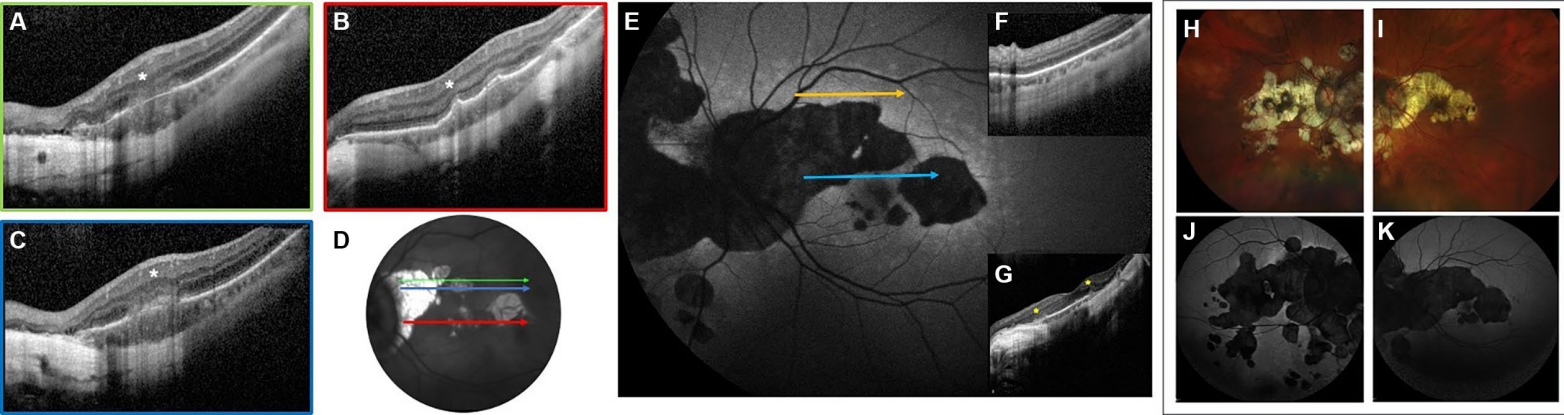
Highly myopic woman (−13 D RE, −11 D LE) who, at the age of 58, consulted for visual disturbances in her LE. Panels **(A–C)** show OCT scans revealing inflammatory lesions and choroidal thickening (asterisks). The fundus image **(D)** indicates the location of each scan; colored arrows correspond to scans framed in the same color. At that time, no specific diagnosis was made, as these findings went unrecognized. Panels **(E–G)** correspond to a subsequent visit at the age of 63, when the patient again consulted for visual disturbances in the LE. FAF **(E)** shows multiple confluent hyperautofluorescent spots surrounding hypoautofluorescent lesions in the posterior pole, consistent with secondary MEWDS. The OCT scan corresponding to the yellow arrow **(F)** shows complete disruption of the ellipsoid zone, while the scan corresponding to the blue arrow **(G)** reveals inflammatory lesions (asterisks). Panels **(H–K)** show current imaging at the age of 67 (**H,I**: retinography; **J,K**: FAF). Notably, the atrophic lesions have enlarged compared with earlier examinations.

**Figure 3:**
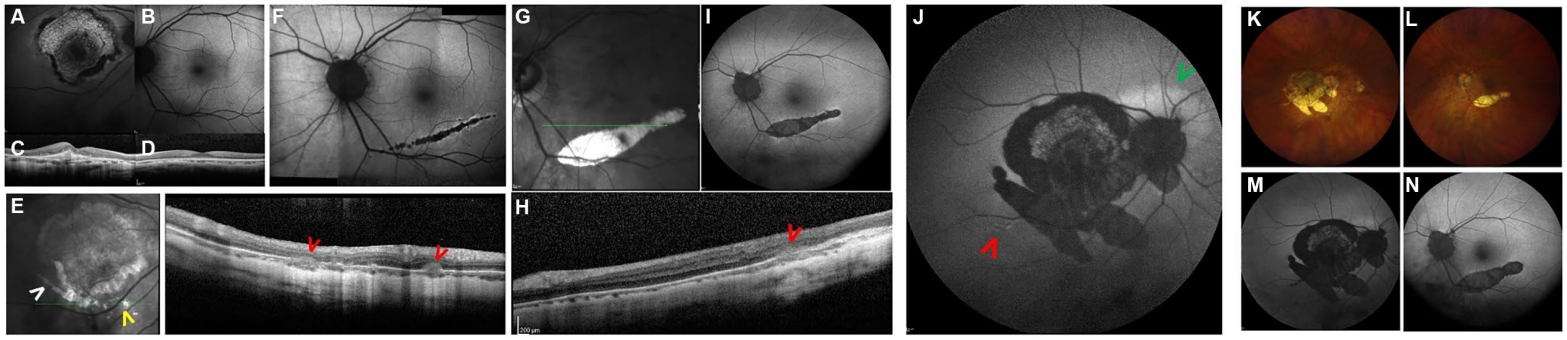
Highly myopic woman (−11 D OU) diagnosed with macular neovascularization (MNV) at the age of 34 and treated with four monthly intravitreal bevacizumab injections. She subsequently developed subretinal fibrosis, and treatment was discontinued. Panels **(A–D)** correspond to a follow-up visit 2 years after the last injection. FAF **(A)** shows a predominantly mixed hyper/hypoautofluorescent area corresponding to fibrosis in the RE, while the LE appears normal **(B)**. OCT scans **(C,D)** show a subretinal hyperreflective lesion in the RE consistent with fibrosis and a normal macula in the LE. Panel **(E)** shows follow-up imaging 3 years after MNV onset. Fundus photography reveals a new patchy atrophic lesion (white arrowhead) and multifocal choroiditis lesions (yellow arrowhead). An OCT scan across these areas demonstrates inflammatory lesions (red arrowheads). Panel **(F)** shows FAF 5 years after MNV diagnosis, when an area of confluent multifocal choroiditis lesions was detected in the asymptomatic LE, appearing as hypoautofluorescent lesions surrounded by a faint hyperautofluorescent halo. Corticosteroids and immunosuppressive therapy were initiated. Panels **(G–I)** show imaging from one of several inflammatory flares during follow-up: infrared imaging and OCT **(G,H)** demonstrate inflammatory lesions (red arrow), and FAF **(I)** shows the development of patchy atrophy at the site of previous inflammatory lesions (see panel **F**). Panel **(J)** shows FAF obtained 12 years after MNV onset, revealing enlargement of patchy atrophy and new acute inflammatory foci in the RE, including small multifocal choroiditis lesions (red arrowhead) and a juxtpapillary hyperautofluorescent area (green arrowhead). Panels **(K–N)** show current imaging at the age of 47 (**K,L**: retinography; **M,N**: FAF), demonstrating further enlargement of atrophic lesions compared with baseline.

Hady et al. (7) explored the relationship between MFC/PIC and PM by analyzing 500 eyes from 253 patients with myopic patchy atrophy using multimodal imaging. They found that 55 eyes (11%) of 39 patients (15.4%) exhibited optical coherence tomography (OCT) features typical of active MFC/PIC lesions, that is focal elevations of the retinal pigment epithelium (RPE) containing homogeneous and medium hyperreflective material, often associated with disruption of the photoreceptor ellipsoid zone and interdigitation zone and choroidal hypertransmission below the lesions. The mean age of affected patients was 57 years, with a mean axial length of 29.2 mm, notably older and more myopic than typical MFC/PIC cohorts (9, 10). Remarkably, 80% of active MFC/PIC lesions evolved into punched out atrophic scars and they tended to enlarge relatively quickly and appeared like patchy atrophy lesions, becoming indistinguishable from myopic patchy atrophy both clinically and on OCT. These findings suggest that a subset of patchy atrophy traditionally attributed to degenerative myopic processes may, in fact, represent the late stage of inflammatory choroiditis. As a matter to remark, macular neovascularization (MNV) was detected in 81.8% of eyes with MFC/PIC, compared with 33.9% in eyes without such lesions, indicating a strong association between inflammatory damage and neovascular complications (odds ratio 8.8; *p* < 0.001). The authors hypothesize that inflammatory disruption of the RPE and Bruch’s membrane, combined with mechanical stretching from myopic axial elongation, may facilitate the development of MNV. Recognizing MFC/PIC-like inflammation in PM is therefore essential, as it carries different therapeutic implications: inflammatory MNV may respond incompletely to anti-VEGF monotherapy and could benefit from adjunctive corticosteroid or immunosuppressive treatment. Overall, this study bridges the gap between inflammatory and degenerative mechanisms in myopic maculopathy and underscores the role of chronic low-grade inflammation in the progression of structural damage in PM. Figures 1–3 illustrate cases of highly myopic patients with patchy atrophy developing at sites of previous inflammatory lesions.

Recent work by Cicinelli et al. (6) has provided compelling structural and temporal evidence that a significant proportion of MFC/PIC lesions represent secondary inflammatory reactions to lacquer cracks, rather than truly idiopathic processes. In their comprehensive observational cohort of 185 eyes with MFC/PIC, the authors demonstrated that 75% of these exhibited a non-random, linear or curvilinear spatial arrangement of lesions, tightly aligned with pre-existing lacquer cracks identified on infrared and indocyanine green angiography. Importantly, the study also established a longitudinal temporal relationship, showing that in fellow eyes initially free of inflammation, lacquer cracks invariably preceded the onset of MFC/PIC lesions, with a median interval of approximately 23 months.

Another relevant contribution of this study is the demonstration that lacquer crack–associated MFC/PIC is characterized by more severe myopia (−11.1 vs. − 5.25 D, *p* < 0.001), thinner choroids, higher lesion burden, and a greater number of inflammatory recurrences than non–lacquer-associated cases. This phenotype appears to reflect a pathophysiological interplay between biomechanical weakening of the RPE–Bruch’s membrane–choriocapillaris complex, immune exposure to previously sequestered antigens, and localized inflammatory activation.

**Figure 4:**
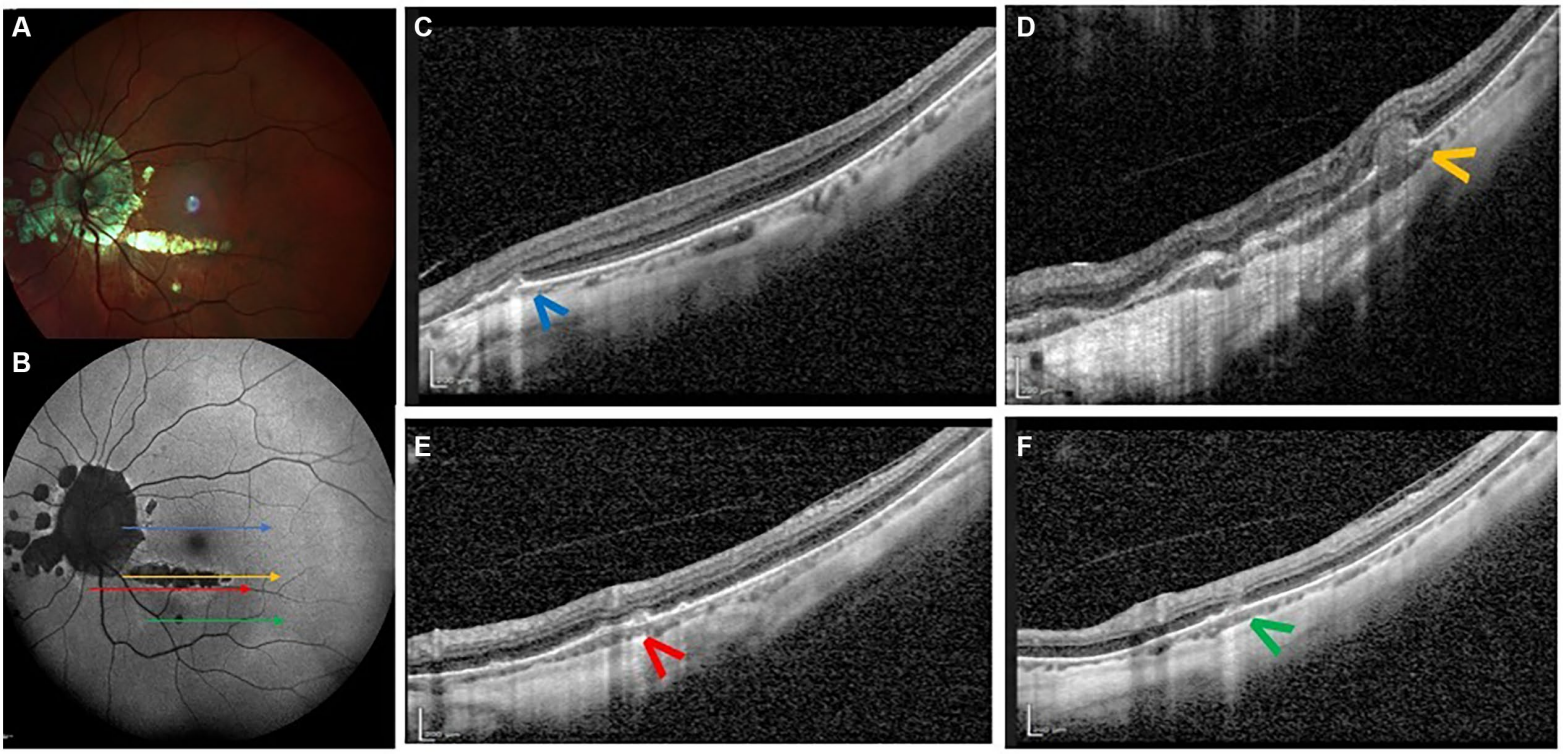
Forty-year-old woman with high myopia (−8 D OU). Panel **(A)** shows multicolor retinography demonstrating patchy atrophy. Panel **(B)** shows FAF revealing hypoautofluorescent lesions around the optic disc and several confluent lesions inferior to the fovea. Panels **(C–F)** show OCT scans obtained at different locations, demonstrating acute inflammatory lesions (colored arrowheads; colors correspond to the matching arrows in the FAF image). The distribution and confluence of lesions within the atrophic area inferior to the fovea suggest that they may have arisen along a pre-existing lacquer crack.

**Figure 5:**
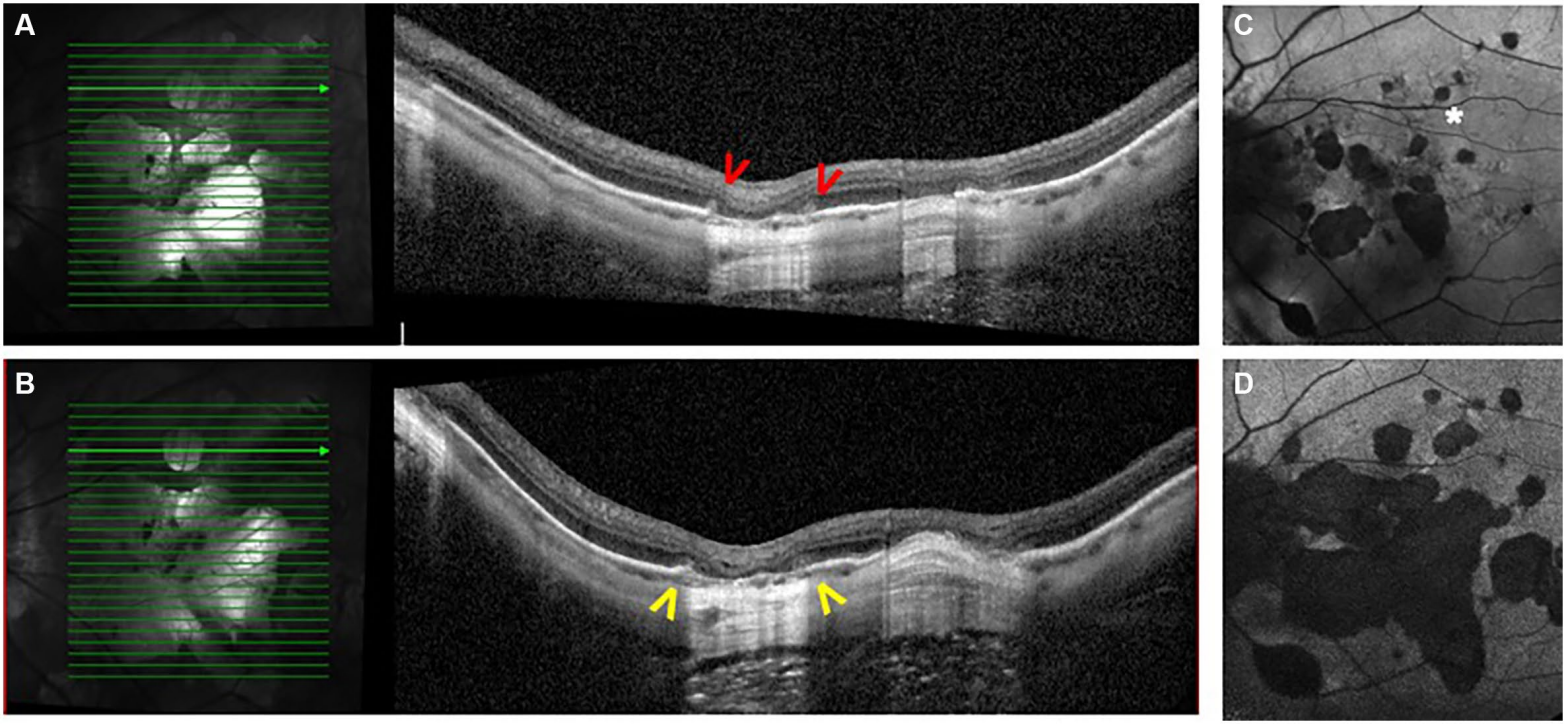
Forty-eight-year-old highly myopic woman (−18 D RE, −13 D LE) followed regularly at the retina clinic. Review of multimodal imaging revealed inflammatory plumes (red arrowheads) on an OCT scan performed in 2020 **(A)**. At the most recent follow-up in 2025, OCT shows expansion of the atrophic areas (yellow arrowheads) **(B)**. Panels **(C,D)** show fundus autofluorescence (FAF) images. Panel **(C)** corresponds to baseline FAF obtained in 2014, whereas panel **(D)** shows FAF at the last follow-up visit in 2025, demonstrating enlargement of the atrophic areas. At baseline, several spots highly suggestive of choroiditis lesions developing along a branched lacquer crack are observed (asterisk).

Figure 4 illustrates a highly myopic patient with patchy atrophy and inflammatory lesions likely arising along a pre-existing lacquer crack. Lesions that may have developed along a lacquer crack are also shown in Figure 5.

## 3 Periatrophic plumes

Ossewaarde-van Norel et al. (8) reported a previously unrecognized form of chronic inflammation in PM, characterized by “periatrophic plumes” of inflammatory material adjacent to areas of macular and peripapillary atrophy. In this retrospective series of 31 eyes from 19 patients followed for an average of 7 years, OCT revealed hyperreflective plumes extending from denuded Bruch’s membrane into the outer retina. The authors describe these periatrophic plumes in two locations: peripapillary atrophy (PPA) (21 eyes) and macular atrophy (MA) (10 eyes). All the eyes that showed expansion of PPA (17 eyes) had plumes, and in 19 eyes where MA expansion occurred, 10 had plumes. Interestingly, the other 9 eyes presented classical inflammatory lesions of MFC, such as inflammatory pigment epithelium detachment, subretinal and outer retinal infiltration by inflammatory cells, and thickened choroid.

According to the authors, the inflammatory origin of the periatrophic plumes is supported by several findings. First, the plumes became noticeably larger during periods of heightened ocular inflammation in other regions. Second, they diminished substantially after corticosteroid therapy—especially when the drug was administered locally—and reappeared once the local corticosteroid effect was presumed to have waned. Third, on OCT, the morphology of the plumes closely resembled that of conventional inflammatory lesions. The inflammatory material was associated not only with loss of RPE but also with direct involvement of Bruch’s membrane. In areas showing acute inflammation, focal defects of Bruch’s membrane were commonly detected. Where the membrane was denuded, accumulations of presumed inflammatory debris were seen adjacent to sites of RPE loss or membrane fragmentation. The authors hypothesize that the initiating trigger may be the exposure of residual fragments of Bruch’s membrane, although inflammatory mediators released from neighboring RPE cells could also contribute to perpetuating the process. These findings suggest that atrophy enlargement in PM is not purely degenerative or myopia-related but may result from persistent, low-grade inflammatory activity at the atrophic borders. Interestingly, the authors comment that they had seen structures like the periatrophic plumes in highly myopic eyes with patchy atrophy who did not have the diagnosis of MFC. Based on this observation, the authors suggest that the pathophysiology of patchy atrophy in HM should be readdressed and inflammatory processes should be considered.

Figure 5 illustrate a case of a highly myopic patient with typical inflammatory plumes.

## 4 Secondary multiple evanescent white dot syndrome (MEWDS)

Based on a literature review and retrospective analysis of their own data, Fouad YA et al. (11) have delineated typical, atypical and secondary MEWDS in a recent publication. Secondary type refers to cases in which MEWDS arises in eyes with previous chorioretinal diseases (12–14). As a matter to remark, PM with lacquer cracks or dome-shaped maculopathy is the second most frequent association after MFC/PIC (11). The underlying hypothesis for secondary MEWDS is that structural damage to the RPE–Bruch’s membrane complex or the outer retina exposes sequestered antigens, triggering a transient immune response that manifests with the typical multimodal imaging features of MEWDS. In PM, progressive mechanical stress, thinning of the choroid, and the formation of lacquer cracks may cause focal breaches in Bruch’s membrane and the RPE, leading to the mentioned antigen exposure and local inflammation. In the above-mentioned study by Cicinelli et al. (6) MEWDS lesions were also observed. They initially appeared along lacquer crack borders and sometimes expanded centrifugally over the following days. In contrast, MFC/PIC lesions development was a slower process taking a median of 2 years to manifest. The authors point out that immune activation would involve different cellular targets in these entities: the photoreceptors in MEWDS and the RPE-Bruch’s membrane-choriocapillaris in MFC/PIC.

It is important to point out that in patients with PM the characteristic white dots of MEWDS may be very difficult to detect on fundoscopy due to tessellated fundus, peripapillary atrophy, or patchy myopic atrophy. FAF is usually the most sensitive modality, showing multiple hyperautofluorescent spots extending beyond the posterior pole. Hence, it is very recommendable to perform FAF (not only OCT) in any highly myopic patient consulting for visual disturbances, especially scotomata.

Secondary MEWDS illustrates the capacity of the myopic eye to generate inflammatory responses secondary to structural stress or tissue damage, rather than through a primary autoimmune or infectious mechanism. In this sense, it may represent an “immune epiphenomenon” of mechanical or ischemic injury, highlighting the dynamic interface between degeneration and inflammation in PM.

**Figure 6:**
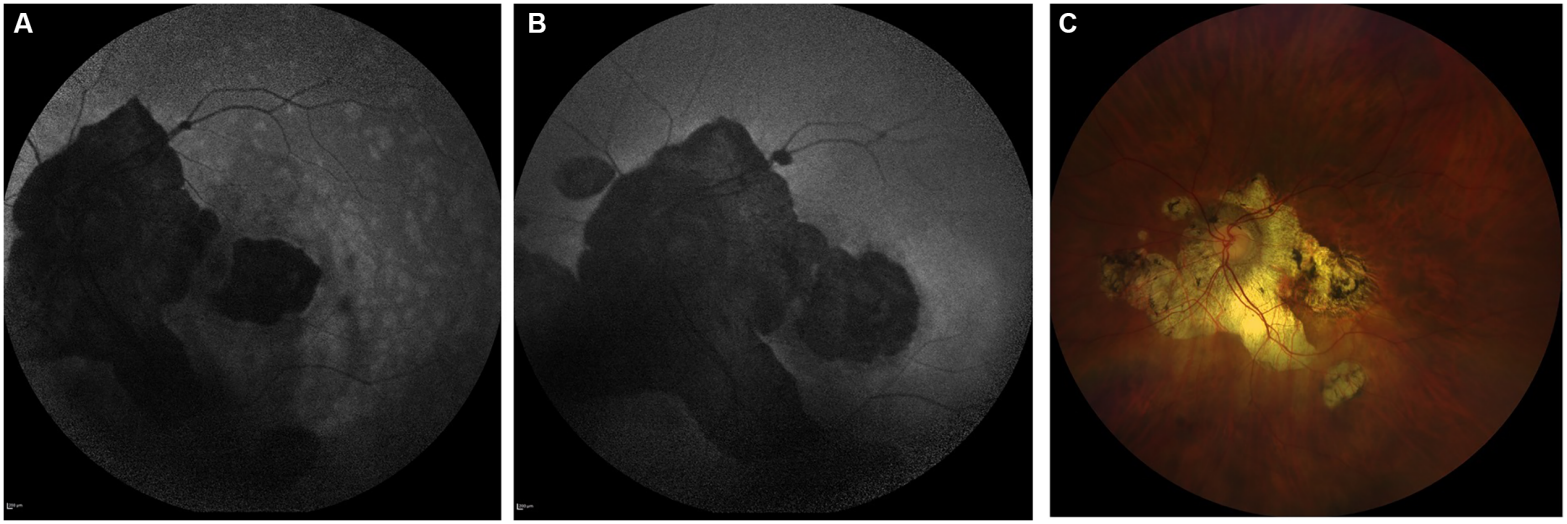
Highly myopic woman (−8 D RE, −13 D LE) who consulted for visual disturbances in the LE at the age of 41. **(A)** Shows FAF revealing multiple hyperautofluorescent spots surrounding atrophic lesions, suggestive of secondary MEWDS. **(B,C)** Show FAF and retinography, respectively, at the most recent follow-up (48 years old).

Figure 6 illustrates a case of secondary MEWDS in a highly myopic patient with patchy atrophy. An additional episode of secondary MEWDS is also shown in Figure 2.

## 5 Discussion

Mounting evidence from both clinical observation and experimental models reinforces the concept that inflammation plays a substantial role in the pathogenesis of PM. Some findings suggest that not only local inflammation, but also systemic inflammation may be involved in this pathogenesis. Significantly higher neutrophil-to-lymphocyte ratios and platelet-to-lymphocyte ratios were found in the peripheral blood of patients with HM (15, 16) and elevations in high-sensitivity C-reactive protein and the complement profile in the peripheral blood of patients with HM were also demonstrated (17). Moreover, it has been noticed that serum concentrations of anti-LIM and senescent Cell antigen-like-containing domain protein 1 (anti-LIMS1) autoantibodies were markedly increased and significantly associated with the severity of myopic macular degeneration (18). Another fact that would support the relation between systemic inflammation and myopia is its higher prevalence in autoimmune diseases like systemic lupus erythematosus or type 1 diabetes (19).

However, local inflammatory factors would probably play a more relevant role. In this regard, aqueous levels of pro-inflammatory cytokines (Chi3l1, IL-6Ra, IL-8, IL-12, IL-27) and inflammation-related cytokines (A proliferation inducing ligand (April), B-cell activating factor (BAFF), IL-34) were increased in patients with more severe forms of PM; however, the anti-inflammation cytokines (IL-11 and aggrecan) decreased progressively with the severity of myopic retinopathy. The JAK–STAT pathway has also been proposed to be involved in the Pathogenesis of PM (20). The complement system has also been pointed out as an important factor. Experimental models of myopia have shown upregulation of complement-related genes in the retina, indicating early complement involvement during myopic remodeling (21). Proteomic studies in patients with PM revealed enrichment of complement and coagulation cascades in the aqueous humor (22). Finally, elevated levels of complement proteins have been found in the aqueous humor of patients with PM (23).

Therefore, it seems that the myopic eye is an inflamed eye. However, the specific role of inflammation in the pathogenic pathway is far from being elucidated. A likely sequence of events can be described as follows: excessive axial elongation in HM arises from progressive remodeling of the sclera and choroid. Under hypoxic stress, scleral fibroblasts release proinflammatory cytokines that activate the matrix metalloproteinase (MMP)-2 signaling pathway, driving fibroblast differentiation, apoptosis, and extracellular matrix degradation, which in turn reduce scleral stiffness and promote axial elongation. Inflammatory mediators further induce cyclooxygenase-2 (COX-2), inducible nitric oxide synthase (iNOS), and additional MMPs in scleral fibroblasts. This sustained activation transforms fibroblasts into modulators and amplifiers of local immune responses, establishing a feedback loop between inflammation and tissue remodeling that ultimately affects the sclera, choroid, and retina (7).

Imaging findings from clinical practice may give us the opportunity to infer some of the potential relationships between inflammation and PM. The study by Cicinelli et al. (6), supports the hypothesis that inflammatory lesions in MFC/PIC arise in lacquer cracks. Therefore, a plausible simplified sequence of events may involve excessive axial elongation leading to Bruch’s membrane rupture and damage to the retina–RPE–choriocapillaris complex, followed by exposure of retinal antigens that trigger inflammatory responses (such as MEWDS or MFC/PIC lesions), facilitated by a pre-existing proinflammatory microenvironment.

Another pathogenic step may be elucidated from the observations by Hady et al. (7) and Ossewaarde-van Norel et al. (8). According to these studies, it becomes plausible that inflammatory activity contributes to the development and the progression of patchy atrophy in PM. The observation that active choroiditis foci or periatrophic inflammatory plumes often precede or accompany expansion of atrophic patches supports a causal rather than incidental relationship, at least in some patients. Moreover, it is conceivable that similar inflammatory events may also take place in a larger proportion of patients. These acute episodes could easily go unnoticed and not objectivated by multimodal imaging, since they may be asymptomatic or though symptomatic, patients may not consult. Continuous or high-frequency imaging would be required to capture such fleeting inflammatory activity, which is currently not feasible in clinical practice. A provocative and intriguing hypothesis would be that inflammatory phenomena are the main driving force behind atrophic expansion in all patients with PM. In this view, inflammation would represent a universal, though variably expressed, mechanism that governs the transition from structural vulnerability to irreversible tissue loss. While such a hypothesis remains impossible to prove with current imaging frequency and sensitivity, it offers a compelling framework to reinterpret the pathogenesis of myopic patchy atrophy through an inflammatory lens. Nevertheless, the current evidence remains circumstantial. Most human data derive from cross-sectional or retrospective studies, and it is still uncertain whether inflammation is the initiating cause of atrophy or a secondary response to tissue damage and oxidative stress. Future research should involve both clinical and experimental perspectives. Longitudinal imaging and biomarker studies are required to establish temporality. To our knowledge, prospective evaluation of degenerative and inflammatory lesions in MFC/PIC patients is limited to a subgroup of 15 patients in the study by Cicinelli et al. (6) in whom lacquer cracks preceded the development of MFC/PIC lesions. Cohorts with larger number of patients are needed to confirm these observations as well as to understand the dynamics, behavior and mechanisms of inflammatory flares leading to enlargement of atrophy, which may result from confluence of different inflammatory foci or from repetitive inflammatory foci at the margin of an atrophic lesion or within the lesion itself (24). There are also some other issues that need to be addressed in future investigations like the development of MFC/PIC lesions in myopic eyes without lacquer cracks, or more intriguingly, in eyes without myopia and thickened choroids, in which choroidal hyperpermeability could underlie the pathogenesis of RPE-Bruch’s membrane damage (25). Genetic background may play a role at different levels and could explain, for instance, why not all lacquer cracks develop inflammatory foci. Finally, of particular interest is the strong association between MFC/PIC and female sex, which may reflect a hormone related proinflammatory state, as happens with systemic autoimmune diseases.

Experimental models combining induced axial elongation with controlled mechanical disruption of Bruch’s membrane or *in vitro* studies using scleral fibroblasts and RPE cells exposed to mechanical stress or hypoxia may help determine how structural abnormalities trigger secondary inflammatory responses in the outer retina. Finally, advanced ex vivo and organoid-based retinal models may provide additional insight into inflammatory signaling following focal RPE–Bruch’s membrane injury.

Understanding these interactions, that is the self-perpetuating feedback loop “degeneration-inflammation- degeneration” will be essential to determine whether controlling inflammation can indeed slow the structural and functional decline in PM. At present, with the currently available data, a reasonable approach for the management of patients with patchy atrophy in which acute inflammatory lesions (typical MFC/PIC lesions, plumes or even secondary MEWDS) have been previously objectivated by means of OCT and or FAF, might be to assume the expansion of the atrophy as an inflammation-related growth, and hence, consider just in these cases treatment with immunosuppressive agents.

In summary, increasing clinical and experimental evidence supports a close interplay between inflammation and structural remodeling and damage that underlies PM. The key points that arise from multimodal imaging findings, and that might be relevant in the pathogenesis of PM in at last a subgroup of patients, are: 1. Inflammatory foci occurrence in lacquer cracks; 2. Secondary focal chorioretinal atrophy formation after resolution of inflammation; 3. The confluence of different foci contributes to the formation of patchy atrophy, as well as the growing of individual atrophic lesions, in some instances due to recurrent/chronic inflammation at the margins or in the lesion itself.

Future longitudinal and molecular studies are needed to clarify these concepts and to identify patients who may benefit from anti-inflammatory or immunomodulatory treatment aimed at slowing atrophy expansion.
